# Gallstone-Induced Perforation of the Common Bile Duct in Pregnancy

**DOI:** 10.1155/2008/174202

**Published:** 2008-06-30

**Authors:** N. Dabbas, M. Abdelaziz, K. Hamdan, B. Stedman, M. Abu Hilal

**Affiliations:** ^1^Department of Surgery, Southampton General Hospital, Tremona Road, Southampton, Hampshire SO16 6YD, UK; ^2^Department of Radiology, Southampton General Hospital, Tremona Road, Southampton, Hampshire SO16 6YD, UK

## Abstract

Spontaneous perforation of the extrahepatic biliary system is a rare presentation of ductal stones. We report the case of a twenty-year-old woman presenting at term with biliary peritonitis caused by common bile duct (CBD) perforation due to an impacted stone in the distal common bile duct. The patient had suffered a single herald episode of acute gallstone pancreatitis during the third trimester. The patient underwent an emergency laparotomy, bile duct exploration, and removal of the ductal stone. The postoperative course was uneventful.

## 1. INTRODUCTION

Perforation of the biliary system occurs most frequently in the gallbladder, usually associated with (and complicating upto 10% cases of) acute cholecystitis. Perforation of the extrahepatic biliary tree is a rare entity, accounting for less than 10% of intraperitoneal biliary rupture [1].

Bile duct perforation is most commonly described in infants related to congenital biliary system anomalies. Aetiology in the adult is commonly attributable to intramural infection, necrosis of the wall of the bile duct secondary to thrombosis, increased intraductal pressure secondary to obstruction, cirrhosis, and direct erosion by calculi. Overall, 70% of cases are related to calculi [2].

The incidence of biliary tract disease during pregnancy ranges from 0.05–0.3% [3]. Despite these apparent low figures, complications from gallstones represent the most common general surgical condition requiring surgical intervention, second only to appendicitis [4]. Indications for intervention of gallstones during pregnancy include obstructive jaundice, acute cholecystitis, or pancreatitis failing medical management. We present the case of a young woman diagnosed with gallstones in late pregnancy, complicated by acute gallstone pancreatitis and subsequently spontaneous common bile duct perforation.

## 2. CASE PRESENTATION

A twenty-year-old primigravida woman was planned for elective caesarean section due to breech presentation. The patient had a past medical history of *α*-Thalassemia trait, but was not normally on regular medication. Her mother had previously undergone a cholecystectomy for gallstones.

At 34 weeks gestation, she presented acutely with a two-week history of worsening abdominal pain localised to the epigastric region, associated with vomiting. On examination, tenderness was localised to the epigastrium and right upper quadrant. Blood results revealed raised inflammatory markers (WBC 14.4 [4.0–11.0], neutrophils 11.9 [2.0–7.5], CRP 60 [0–7.5]) and evidence of pancreatitis (amylase 1369 IU/L [36–128]), mildly raised bilirubin (24 *μ*mol/L [0–20]) and raised alkaline phosphatase (183 IU/L [35–91]). An abdominal ultrasound revealed multiple small gallstones and a thickened gallbladder wall, but no evidence of a dilated intra or extrahepatic biliary system. The patient was treated conservatively, rapidly improved, and liver function tests normalised. She was discharged three days later.

Six weeks later, she was readmitted with severe abdominal pain. She was pyrexial (39.3°C), tachycardic, and hypotensive. Abdominal examination revealed severe generalised abdominal tenderness with evidence of peritonism. An emergency caesarean section was performed and a term baby delivered, but no obvious cause was found to explain her clinical condition.

**Figure 1:**
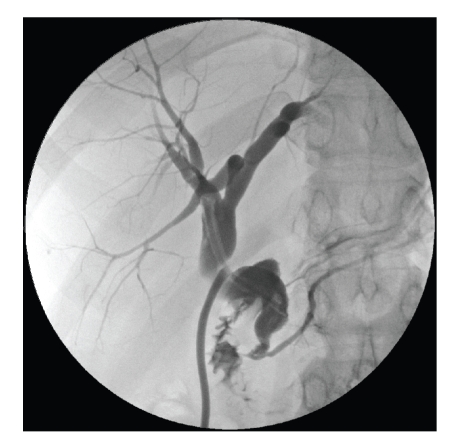
Postoperative T-tube cholangiogram illustrating normal duodenal filling with no visible bile duct filling defect.

The following day her clinical condition worsened, with progressive abdominal pain and a metabolic acidosis. She required aggressive resuscitation, inotropic, and ventilatory support and was, therefore, admitted to the intensive care unit. A computed tomography (CT) revealed extensive free peritoneal fluid and gas of which the aetiology was not apparent. The patient underwent a prompt laparotomy and was found to have generalised biliary peritonitis. The gallbladder was intact but a 2 mm perforation was found on the anterior surface of a dilated common bile duct (12 mm). On table cholangiography suggested obstruction of the distal common bile duct caused by a 5 mm gallstone impacted within the distal common bile duct. The calculus was removed, and the duct was repaired over a T-tube. Repeat cholangiography showed no residual obstruction. The patient had an uneventful postoperative recovery and was discharged on day seven. A T-tube cholangiogram was performed after 4 weeks, and the tube was uneventfully removed (Figure 1).

## 3. DISCUSSION

Although the pathogenesis of spontaneous biliary perforation is poorly understood, recognised mechanisms include the following: calculous perforation at the site of impaction; calculous erosion without impaction; increased canalicular pressure due to obstruction by tumour, stone, or spasm of the sphincter of Oddi; intramural infection; mural vessel infarction leading to mural necrosis; or rupture of a biliary tract anomaly such as cyst or diverticulum [5]. Thus, because perforation of the biliary system is a recognised complication of cholelithiasis, the diagnosis should be suspected if a perihepatic abscess or peritonitis is combined with biliary stone disease.

As early as 1882, Freeland [6] reported the first case of extrahepatic biliary system rupture in an adult (diagnosed at autopsy), an entity that was subsequently first described in pregnancy by Piotrowski et al. [2] over a century later. Since this time, very few cases of spontaneous common bile duct perforation in adults have been reported in the literature, with cases occurring during pregnancy being even more scarce. The importance of this clinical scenario lies in the potential serious morbidity and not infrequent mortality associated with missed biliary system perforation.

Petrozza et al. [7] described two cases of gallbladder perforation due to cholelithiasis in the early postpartum period. Both cases presented a diagnostic dilemma, and it was concluded that a history of cholelithiasis in a patient with persistent intra-abdominal symptoms in the postpartum period must alert to prompt investigation and early management. Talwar et al. [8] report two cases of biliary tract rupture under similar circumstances. In their series, one patient was found to have suffered gallbladder rupture as a result of cholecystitis, and in the second, a common bile duct perforation was found at laparotomy with no obvious precipitating cause. McGrath et al. [9] also drew attention to the similarity of symptoms of gallbladder disease in pregnancy to mild pre-eclampsia, having in common hypertension, epigastric pain, and mildly deranged liver function tests. These cases highlight the importance of recognising the possibility of delayed diagnosis of cholelithiasis as a result of nonspecific abdominal symptoms during pregnancy and indicate early investigation and treatment in order to reduce serious morbidity.

Several reports exist of successful laparoscopic cholecystectomy during pregnancy. Block and Kelly [10] reported the optimum time for surgical management of gallstone pancreatitis to be in the second trimester or early postpartum period, in order to minimise maternal/fetal mortality and recurrent pancreatitis.

Unfortunately, in those women presenting late in pregnancy (as in the case described), the balance of risk favours watchful waiting until after delivery followed by elective cholecystectomy. Certainly, this risks early recurrence of acute pancreatitis, as well as rare but severe consequences such as biliary peritonitis. Whether an early endoscopic retrograde cholangiopancreaticography (ERCP) and sphincterotomy in those cases presenting with gallstone pancreatitis can be an acceptable temporary preventive measure is unclear, but undertaking ERCP is not without risk, and the potential risks should be considered carefully in individual cases. In this particular case, it is impossible to know whether the eroding calculus had been present during the initial episode of pancreatitis. Magnetic resonance scanning is a commonly used imaging modality in obstetrics, considered to be safe and avoiding the use of ionising radiation. Therefore, magnetic resonance cholangiopancreatography (MRCP) would have been a reasonable next investigation during this patient’s initial presentation, and if a ductal stone had been revealed, then the indication for ERCP may have been clearer.

On the other hand, neonatal and postnatal care of babies born early have progressed significantly, suggesting the possibility of induction of labour perhaps at 36–38 weeks gestation in severely symptomatic or high-risk patients. Of course, every case must be considered individually, taking into account maternal and fetal history and health.

## References

[1] McWilliams CA (1912). Acute spontaneous perforation of the biliary system into the free peritoneal cavity. *Annals of Surgery*.

[2] Piotrowski JJ, Van Stiegmann G, Dale Liechty R (1990). Spontaneous bile duct rupture in pregnancy. *HPB Surgery*.

[3] Lu EJ, Curet MJ, El-Sayed YY, Kirkwood KS (2004). Medical versus surgical management of biliary tract disease in pregnancy. *The American Journal of Surgery*.

[4] Printen KJ, Ott RA (1978). Cholecystectomy during pregnancy. *The American Surgeon*.

[5] Rege SA, Lambe S, Sethi H, Gandhi A, Rohondia O (2002). Spontaneous common bile duct perforation in adult: a case report and review. *International Surgery*.

[6] Freeland J (1882). Rupture of the hepatic duct. *The Lancet*.

[7] Petrozza JC, Mastrobattista JM, Monga M (1995). Gallbladder perforation in pregnancy. *American Journal of Perinatology*.

[8] Talwar N, Andley M, Ravi B, Kumar A (2006). Spontaneous biliary tract perforations: an unusual cause of peritonitis in pregnancy. Report of two cases and review of literature. *World Journal of Emergency Surgery*.

[9] McGrath BA, Singh M, Singh T, Maguire S (2005). Spontaneous common bile duct rupture in pregnancy. *International Journal of Obstetric Anesthesia*.

[10] Block P, Kelly TR (1989). Management of gallstone pancreatitis during pregnancy and the postpartum period. *Surgery, Gynecology & Obstetrics*.

